# Multi-Omics Analysis of CDKN2A (p16INK4a) in Cervical Carcinoma in the Context of Human Papillomavirus and in Endometrial Carcinoma

**DOI:** 10.3390/genes17030281

**Published:** 2026-02-27

**Authors:** Rasha Elsayim, Heba W. Alhamdi, Nihal Almuraikhi, Mariam Abdulaziz Alkhateeb, Taghreed Mohamed Osman Derar, Sami Habiballa Abdalla Mohamed, Esra’a Abudouleh

**Affiliations:** 1 Department of Botany and Microbiology, College of Science, King Saud University, Riyadh 11451, Saudi Arabia; 2 Department of Biology, College of Sciences, King Khalid University, P.O. Box 960, Abha 61413, Saudi Arabia; 3 Stem Cell Unit, Department of Anatomy, College of Medicine, King Saud University, Riyadh 11461, Saudi Arabia; nalmuraikhi@ksu.edu.sa; 4 Department of Biology, College of Science, Princess Nourah bint Abdulrahman University, P.O. Box 84428, Riyadh 11671, Saudi Arabia; 5 Health Sciences Department, College of Applied Studies, King Saud University, Riyadh 12372, Saudi Arabiasamabdalla@ksu.edu.sa (S.H.A.M.)

**Keywords:** CDKN2A, cervical squamous cell carcinoma, endometrial carcinoma, human papillomavirus, transcriptomic and proteomic evidence

## Abstract

Background: CDKN2A (p16^INK4a^) is integral to the regulation of the RB–E2F cell-cycle checkpoint and is widely acknowledged as a surrogate marker for high-risk human papillomavirus (HPV)-related cervical neoplasia. Nevertheless, its diagnostic and prognostic significance in uterine corpus endometrial carcinoma (UCEC), a predominantly HPV-independent malignancy, remains inadequately characterized. This study utilized an integrated multi-omics approach to examine CDKN2A dysregulation in cervical squamous cell carcinoma (CESC) and UCEC. Methods: Pan-cancer and tumor–normal differential expression analyses were performed using TIMER2.0 and GEPIA2 (TCGA/GTEx). Clinicopathological correlations were assessed with UALCAN. Protein expression patterns were analyzed using immunohistochemistry data from the Human Protein Atlas (HPA). Prognostic significance and immune-infiltration associations were evaluated using TCGA survival data and TIMER modules. Independent transcriptomic validation and diagnostic classification performance were assessed using GEO datasets GSE9750 (CESC) and GSE63678 (UCEC), including ROC-AUC analysis with cross-validation. Results: Integrated analyses revealed elevated CDKN2A expression in both CESC and UCEC across multiple transcriptomic cohorts, with pronounced tumor-specific protein expression on immunohistochemistry. TCGA-only tumor–normal RNA comparisons were non-significant, likely due to limited normal sample representation. In independent GEO cohorts, CDKN2A exhibited excellent tumor–normal discrimination in CESC (AUC = 0.982) and moderate discrimination in UCEC (AUC = 0.761). Survival analysis indicated tumor-specific patterns, with limited prognostic stratification in CESC and context-dependent associations in UCEC. Immune-infiltration analysis suggested tumor-type-specific interactions between CDKN2A expression and immune cell subsets. Conclusions: CDKN2A exhibits strong diagnostic performance in HPV-associated cervical cancer and moderate, cohort-dependent discriminatory ability in endometrial carcinoma. These findings reinforce its established diagnostic role in CESC and propose adjunctive utility in UCEC, underscoring the importance of tumor-contextual interpretation of CDKN2A expression in gynecologic malignancies.

## 1. Introduction

Cervical squamous cell carcinoma (CESC) and uterine corpus endometrial carcinoma (UCEC) are significant gynecological cancers with different causes but a considerable impact on global health. Cervical cancer poses a major public health issue, with approximately 604,127 new cases and 341,831 deaths worldwide in 2020, translating to an age-standardized incidence rate of 13.3 per 100,000 women [1]. The disease burden is unevenly distributed, with the highest rates of incidence and mortality in low-resource areas, especially in sub-Saharan Africa, highlighting disparities in access to vaccination, screening, and treatment services [2] Data on global distribution further reveal that cervical cancer incidence and mortality rates decrease as the Human Development Index increases, indicating a clear socioeconomic gradient in disease burden [1]. Regional statistics show significant variability, with some African nations having the highest incidence rates globally, underscoring stark international differences in disease risk and outcomes [1]. Notably, cervical cancer is largely preventable and treatable if detected early, underscoring the importance of screening and timely diagnosis in reducing mortality [2]. Conversely, UCEC shows a different epidemiological pattern, with rising incidence linked to aging populations and hormonal/metabolic risk factors. In 2020, there were about 417,367 cases and 97,370 deaths from endometrial cancer worldwide, representing roughly 2.2% of all newly diagnosed cancers [3]. The disease burden is greater in developed regions and is closely associated with estrogen exposure and demographic risk factors. Unlike cervical cancer, many UCEC cases are identified early due to symptoms like abnormal uterine bleeding, highlighting the significance of clinical recognition for a favorable prognosis [3]. These epidemiological differences highlight the necessity for molecular biomarkers that can enhance diagnostic stratification and early detection across gynecologic cancers, especially in areas where screening infrastructure is limited [3].

High-risk HPV is the main cause of cervical cancer. It keeps making E6 and E7 proteins from high-risk types like HPV-16/18 [4]. These proteins stop p53 and pRB, which control cell growth, leading to uncontrolled cell growth. These proteins also affect other cell growth signals, making cancer more likely. E6 and E7 also cause DNA changes by skipping cell division checks, helping cancer spread [4]. In contrast, HPV is not a major cause of endometrial cancer (UCEC). A study of 29 studies (1026 cases) showed about 10% had HPV DNA, with no higher risk compared to normal tissue, meaning UCEC is mostly not caused by HPV. UCEC is mainly influenced by hormones and genetic changes, like PTEN, PIK3CA, KRAS, CTNNB1, and FGFR2, which create different types of cancer with specific treatment needs [5]. Because cervical cancer is caused by HPV and UCEC is not, we need tests that work for both types to find and treat them early. In HPV-driven cervical cancer, the E7 protein binds to and breaks down RB, causing p16 to increase, showing RB pathway problems. E7’s effect on RB increases E2F activity and cell growth, matching past findings that HPV16 E7 breaks down RB and activates E2F [5,6]. In practice, p16 testing is a good indicator of high-risk HPV disease about 95% of HPV-related cancers show strong p16 positivity, making it useful in pathology [6]. However, p16 is not completely specific: some HPV-independent cancers also show high p16, which can confuse HPV-related diagnosis [4]. In practice, p16 testing is a standard tool for HPV-related lesions, with studies showing over 99% of CIN3 cases are p16-positive, while benign samples rarely are [7]. In contrast, UCEC is mostly not caused by HPV, with a study showing about 10% HPV DNA presence and no higher risk compared to normal tissue, so p16’s value in UCEC is unclear [6]. Given these differences and p16’s imperfect specificity, we need tests that work for both HPV and non-HPV gynecologic tumors, with strong test performance.

CDKN2A is markedly and consistently overexpressed in cervical cancers associated with HPV, a result of RB inactivation by E7 and the subsequent compensatory rise in p16 levels [4]. Therefore, a thorough multi-omics analysis that integrates transcriptomic, proteomic, and clinical outcome data is essential to clarify CDKN2A dysregulation across gynecologic cancers and to determine if UCEC exhibits a pattern similar to HPV-driven tumors [4]. This research aimed to determine the diagnostic and prognostic relevance of CDKN2A (p16^INK4a^) in HPV-associated cervical cancer and endometrial carcinoma through an integrated approach involving transcriptomic and proteomic analyses. Recognizing the role of HPV in inducing CDKN2A expression in cervical neoplasia, we investigated whether this molecular signature is similarly present in endometrial cancer by analyzing datasets from TCGA, GEPIA, UALCAN, HPA, and GEO. Our analyses focused on differential expression, clinicopathological associations, immune infiltration, survival outcomes, and cross-validation using GSE9750 and GSE63678. We suggested that HPV-driven dysregulation results in CDKN2A (p16^INK4a^) overexpression in cervical cancer and hypothesized that this pattern is also evident in endometrial carcinoma, thereby reinforcing its potential as a diagnostic biomarker. Additionally, we anticipated that CDKN2A expression levels would be linked to survival outcomes and variations in the immune microenvironment in a manner specific to the tumor type.

## 2. Materials and Methods

### 2.1. Study Design and Data Sources

This investigation adopted a robust multi-omics bioinformatics strategy to explore the diagnostic and prognostic implications of CDKN2A (p16^INK4a^) in cervical squamous cell carcinoma (CESC) and uterine corpus endometrial carcinoma (UCEC), with a specific focus on the regulation mediated by human papillomavirus (HPV) in cervical cancer. The study utilized publicly accessible datasets and analytical tools, including: TIMER 2.0 for pan-cancer expression profiling, GEPIA 2.0 for differential expression analysis between tumor and normal tissues (TCGA/GTEx), UALCAN for clinicopathological subgroup analysis, Human Protein Atlas (HPA) for immunohistochemistry and RNA expression data, TCGA (FPKM-UQ) for transcriptomic and clinical outcome data, and GEO datasets for independent validation: GSE9750 (CESC) and GSE63678 (UCEC) [8].

### 2.2. Pan-Cancer Screening

The study of pan-cancer differential expression was executed using TIMER 2.0 (https://timer.cistrome.org/, accessed on 21 September 2025), which offers visualization of log2(TPM + 1)-normalized RNA-seq expression profiles between tumor and adjacent normal tissues, employing the Wilcoxon rank-sum test for analysis. Following this, GEPIA 2.0 (TIMER, https://cistrome.shinyapps.io/timer/, accessed on 24 September 2025) was utilized to confirm tumor-specific expression patterns by integrating TCGA tumor samples with GTEx normal tissues. The analyses adhered to log2(TPM + 1) normalization, with statistical significance determined by thresholds of |log2FC| > 1 and q < 0.05.

### 2.3. Clinicopathological Correlation

UALCAN (http://ualcan.path.uab.edu, accessed on 28 September 2025) served as the analytical tool to examine CDKN2A expression across distinct clinicopathological subgroups, which included various age brackets, racial demographics, tumor stages (I–IV), and a comparison between tumor and normal tissue samples. The platform employs TCGA level-3 RNA-seq data, which is normalized to transcripts per million (TPM) and subjected to log2 transformation. The platform’s implementation of Student’s *t*-test facilitates group comparisons, and the sample sizes for each subgroup were directly extracted from the UALCAN output panels. The significance levels reported are aligned with the platform’s predefined thresholds (* *p* < 0.01, ** *p* < 0.001, *** *p* < 0.0001).

### 2.4. Protein-Level Validation

Protein and transcript-level validation was conducted using the Human Protein Atlas (HPA; Expression of CDKN2A in endometrial cancer—The Human Protein Atlas; https://www.proteinatlas.org/ENSG00000147889-CDKN2A/cancer/endometrial+cancer#IHC, accessed on 29 September 2025). RNA expression data were obtained from TCGA-derived RNA-seq datasets and reported as normalized expression (NX) values. Immunohistochemical staining images were examined to evaluate nuclear and cytoplasmic p16 (CDKN2A) expression in cervical squamous cell carcinoma and uterine corpus endometrial carcinoma compared with corresponding normal tissues. Antibody-based staining results were reviewed using the validated antibodies HPA073143, CAB000093, CAB000445, and CAB018232 as annotated in the database. Staining annotations were interpreted according to HPA criteria, including intensity classifications (not detected, low, medium, high), quantity of positive cells, and subcellular localization. Representative images were selected to illustrate differential protein accumulation across tissue types [9].

### 2.5. TCGA Transcriptome Verification

Transcript-level validation was executed using TCGA RNA-seq data sourced from the Human Protein Atlas (Expression of CDKN2A in endometrial cancer—The Human Protein Atlas, https://www.proteinatlas.org/ENSG00000147889-CDKN2A/cancer/endometrial+cancer#IHC, accessed on 29 September 2025). Expression values were obtained from FPKM-UQ-normalized datasets and were log2-transformed prior to conducting comparative analyses. The expression levels of CDKN2A were compared between tumor and normal tissues in cohorts of cervical squamous cell carcinoma and uterine corpus endometrial carcinoma. Sample sizes for these comparisons were directly extracted from database outputs and are reported alongside the relevant figures. Statistical differences between groups were determined using the nonparametric Mann–Whitney U test as provided by the platform. Due to the limited number of normal samples available in TCGA-only comparisons for certain cancer types, the results were interpreted with caution, considering the potential impact on statistical power.

### 2.6. Survival and Immune-Infiltration Analysis

Survival stratification based on CDKN2A expression was conducted using a cohort-specific median cutoff, categorizing patients into high- and low-expression groups. Overall survival probabilities were estimated through Kaplan–Meier analysis, with statistical significance evaluated via log-rank testing. Hazard ratios (HRs) with 95% confidence intervals were calculated using Cox proportional hazards regression. Group sizes and statistical outputs were directly obtained from platform-generated outputs. Concurrently, TIMER immune-infiltration modules were employed to assess associations between CDKN2A expression, survival outcomes, and the estimated infiltration of major immune cell populations, including CD8^+^ T cells and macrophages.

### 2.7. GEO Transcriptomic Validation and Differential Expression Analysis

Independent validation was conducted utilizing publicly accessible microarray datasets from the Gene Expression Omnibus (GEO), specifically GSE9750 (cervical squamous cell carcinoma; GPL96 platform) and GSE63678 (uterine corpus endometrial carcinoma; GPL571 platform). The final cohort analyzed for GSE9750 comprised 65 samples, including 41 tumor and 24 normal samples. For GSE63678, the cohort consisted of 35 samples, with 18 tumor and 17 normal samples. Expression matrices and phenotype annotations were directly retrieved from GEO using GEO parse in Python version 3.12.12. Tumor and normal samples were stringently defined based on the metadata labels in the “title” field of each dataset, where samples labeled with terms such as “tumor,” “carcinoma,” or similar were classified as tumor (coded as 1), and those labeled “normal” or “control” were classified as normal (coded as 0). As the GEO series matrices contained probe-level summarized expression values, no further normalization was applied beyond the original platform preprocessing. CDKN2A probe mapping was executed using platform annotation tables (GPL96 and GPL571). In instances where multiple probes corresponded to CDKN2A, the probe exhibiting the highest variance across samples was selected to represent gene-level expression. Differential expression between tumor and normal groups was evaluated using linear modeling. Log_2_ fold change (log_2_FC) was calculated as follows: Statistical significance was assessed using two-sided *t*-tests. To account for multiple hypothesis testing across all genes, *p*-values were adjusted using the Benjamini–Hochberg false discovery rate (FDR) procedure. Genes with adjusted *p*-values less than 0.05 were deemed statistically significant. Volcano plots were generated to illustrate differential expression patterns. The complete computational pipeline is provided in Supplementary File S1 [10].

### 2.8. Diagnostic Classification Performance Analysis

**Figure 1:**
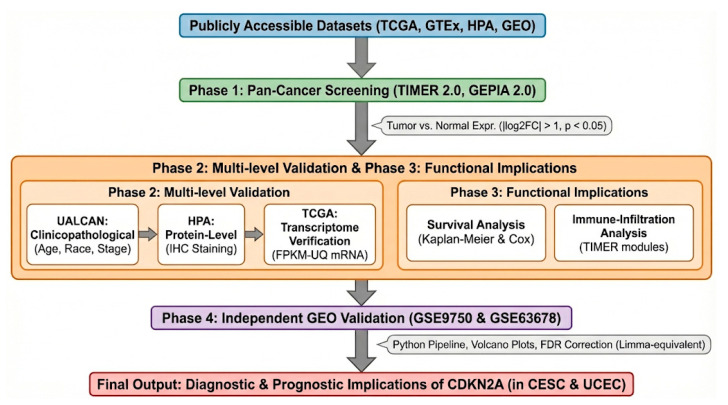
Overview of the multi-omics analytical workflow used to investigate CDKN2A in gynecologic cancers. Public datasets were screened for differential expression, validated across clinicopathological, protein, and transcriptomic levels, followed by survival and immune-infiltration analyses and independent GEO validation, culminating in integrated diagnostic and prognostic interpretation.

To assess the discriminatory capability between tumor and normal samples, CDKN2A expression was analyzed using receiver operating characteristic (ROC) analysis across two independent GEO cohorts. A single-feature classification approach was employed, utilizing stratified 5-fold cross-validation to mitigate overfitting. Within each fold, expression values were standardized through z-score normalization based on the training set. Out-of-fold predicted scores were compiled to construct cross-validated ROC curves. The area under the ROC curve (AUC) served as the primary metric for diagnostic discrimination. To evaluate statistical uncertainty, 95% confidence intervals (CIs) were calculated using non-parametric bootstrap resampling with 2000 iterations. Optimal classification thresholds were identified using the Youden index (sensitivity + specificity − 1). Sensitivity, specificity, and balanced accuracy were reported at the Youden-optimal threshold. All analyses were conducted in Python utilizing scikit-learn (v1.4.2), NumPy, and pandas within a Google Colab environment. The overall analytical workflow of the multi-omics bioinformatics strategy is illustrated in Figure 1.

### 2.9. Statistics and Ethical Statement

Significance thresholds included |log2FC| > 1 and adjusted *p* < 0.05. Survival was assessed using the log-rank test and Cox proportional hazards model [11,12]. Ethical approval was waived in accordance with World Medical Association’s Declaration of Helsinki, as this study was based solely on bioinformatics analysis of publicly available, anonymized datasets and did not involve direct interaction with human participants or access to identifiable personal data.

## 3. Results

**Figure 2:**
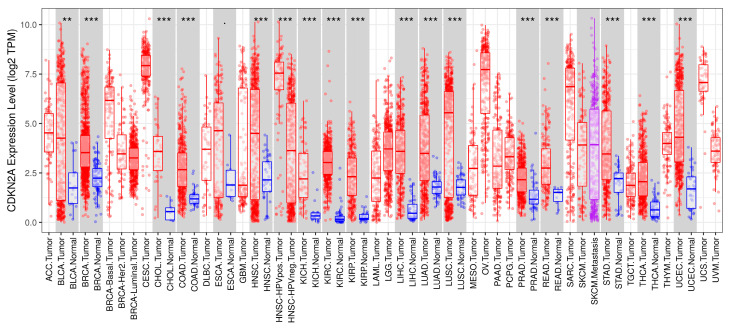
CDKN2A expression profile across different cancer types using the TIMER database. Distributions of CDKN2A expression levels are displayed using box plots; red box plots mean that CDKN2A is up regulated, and blue box plots mean it is down-regulated (**: *p*-value <0.001; ***: *p*-value < 0.0001).

The first set of analyses examined expression profile of CDKN2A across different cancer types using the TIMER database (TIMER, https://cistrome.shinyapps.io/timer/, accessed on 21 September 2025). The expression of CDKN2A was significantly elevated in tumor tissues compared to matched normal tissues across the majority of cancer types. As illustrated in Figure 2, CDKN2A (log2 TPM) levels were upregulated in most TCGA tumor cohorts, including BLCA, BRCA, CESC, CHOL, COAD, ESCA, GBM, HNSC, KIRC, KIRP, LGG, LIHC, LUAD, LUSC, PAAD, PRAD, READ, SARC, STAD, THCA, and UCEC, with *p*-values ranging from 0.01 to 0.0001. Only a limited number of cancers exhibited no significant difference in expression between tumor and adjacent normal tissues. Across multiple cohorts, boxplots and jitter distributions consistently demonstrated a higher median CDKN2A expression in tumors (red) compared to normal tissues (blue), indicating its frequent dysregulation in malignancy.

To validate the expression pattern of CDKN2A observed in TIMER across similar cancer types, we further analyzed data using the GEPIA database (http://gepia.cancer-pku.cn, accessed on 21 September 2025). The results, presented in Supplementary Figure S1, revealed that CDKN2A is significantly upregulated in multiple cancer types when comparing tumor tissues with their matched normal controls. Significant overexpression was observed in BLCA, BRCA, CESC, COAD, HNSC, KICH, KIRC, LIHC, LUAD, LUSC, PRAD, READ, STAD, THCA, and UCEC, as indicated by *p* < 0.05. For each of these cancers, tumor samples exhibited a noticeably higher median CDKN2A expression level, highlighting a consistent dysregulation pattern across diverse tumor types. This finding supports the potential role of CDKN2A in tumorigenesis and malignant progression across multiple solid cancers. After filtering the GEPIA dataset to exclude cancer types with downregulated CDKN2A expression, a refined panel of tumors was identified that consistently demonstrated significant CDKN2A overexpression compared with normal tissues. The final set of up-regulated cancers included BLCA, BRCA, CESC, COAD, HNSC, KICH, KIRC, KIRP, LIHC, LUAD, LUSC, READ, STAD, and UCEC, all showing a marked elevation in CDKN2A levels (*p* < 0.05). As illustrated in Supplementary Figure S2, tumor samples in these cancer types exhibited a noticeably higher median CDKN2A expression relative to matched normal tissues, confirming a recurrent pattern of CDKN2A activation across multiple solid tumors. This refinement step further strengthens the evidence that CDKN2A upregulation is a common molecular feature in malignancies with proliferative and cell-cycle dysregulation.

**Figure 3:**
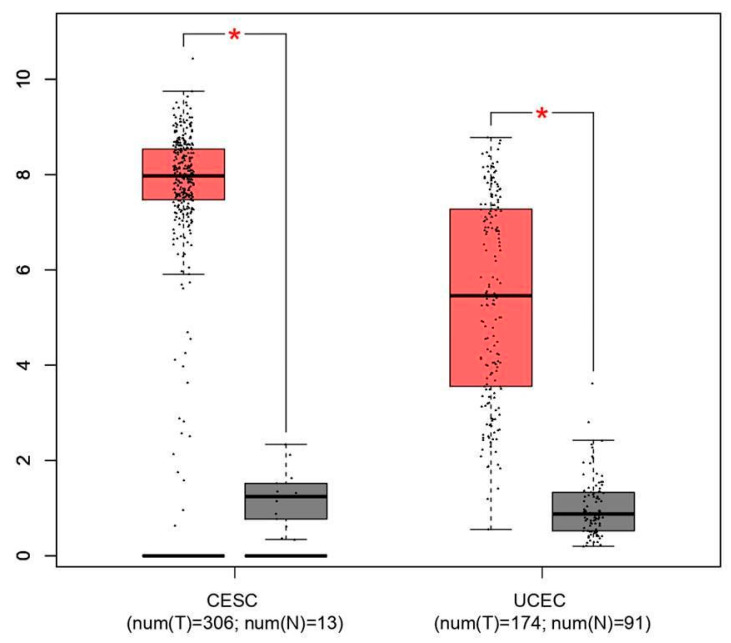
Gene expression among the significant cancer types using the GEPIA database. Each box plot represents the expression of CDKN2A; red box plots refer to up-regulation, and grey box plots represent down regulation. * indicates statistical significance (*p* < 0.05).

Supplementary Figure S3 illustrates Kaplan–Meier survival analyses comparing early- and late-stage patient cohorts, generated using refined GEPIA filtering parameters. The analysis revealed no statistically significant difference in survival between the stage groups (log-rank *p* = 0.57). This outcome is likely attributable to limited statistical power due to the small sample size of late-stage cases, rather than the absence of established stage-dependent prognostic effects. Consequently, this exploratory comparison was not utilized to inform tumor-type prioritization. Instead, the selection of cervical squamous cell carcinoma (CESC) and uterine corpus endometrial carcinoma (UCEC) for subsequent analyses was predicated on consistent CDKN2A overexpression identified through integrated differential expression analyses. Both cancer types exhibited clear tumor–normal expression separation (*p* < 0.05; Figure 3), thereby supporting their suitability for biomarker-focused investigation.

**Figure 4:**
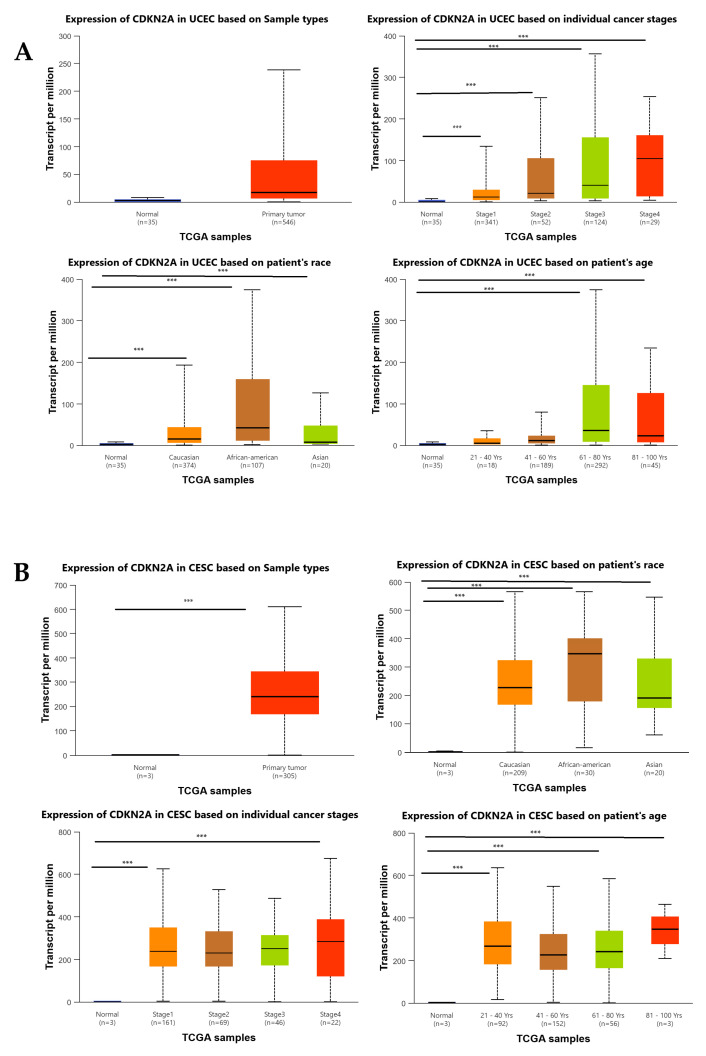
CDKN2A expression analysis based on TCGA data using the UALCAN database. (**A**) CDKN2A expression in cervical squamous cell carcinoma (CESC) according to sample type (normal vs. tumor), individual cancer stages (Stage I–IV), patient race (Caucasian, African American, and Asian), and age groups (21–40, 41–60, 61–80, and 81–100 years). (**B**) CDKN2A expression in uterine corpus endometrial carcinoma (UCEC) according to sample type (normal vs. tumor), individual cancer stages (Stage I–IV), patient race (Caucasian, African American, and Asian), and age groups (21–40, 41–60, 61–80, and 81–100 years). Statistical significance is indicated as follows: *** *p* < 0.0001.

To further investigate the dysregulation of CDKN2A in gynecologic cancers, we examined its expression in UCEC and CESC using UALCAN, taking into account various clinical and demographic factors (Figure 4A,B). In both cancer types, CDKN2A expression was significantly higher in primary tumor samples compared to normal tissues (*p* < 0.0001), reinforcing its strong link to malignant transformation. In UCEC (Figure 4A), CDKN2A expression showed a progressive increase with advancing tumor stages, with significantly elevated levels in stages 1–4 compared to normal endometrial tissue (*p* < 0.0001). Age stratification revealed a clear age-dependent expression pattern, with individuals aged 61–80 years and 81–100 years exhibiting the highest CDKN2A levels (*p* < 0.0001). Additionally, racial comparisons indicated that African American patients had significantly higher CDKN2A expression than Caucasian and Asian groups (*p* < 0.001), suggesting potential molecular differences among ethnic populations. In CESC (Figure 4B), a similar pattern was observed. CDKN2A expression was significantly elevated across all tumor stages compared to normal samples (*p* < 0.001), with consistently high levels from stage 1 through stage 4. Expression also varied significantly among racial groups, with African American patients showing the highest CDKN2A levels, followed by Caucasian and Asian patients (*p* < 0.001). Furthermore, age-group analysis revealed that tumor tissues from patients aged 21–40, 41–60, and 61–80 years all exhibited significantly higher CDKN2A expression than normal cervix samples (*p* < 0.0001). Overall, these UALCAN findings indicate that CDKN2A overexpression is not only tumor-specific but also associated with key clinical and demographic factors, including tumor stage, age, and race, highlighting its potential as a biologically relevant diagnostic and prognostic biomarker in both UCEC and CESC.

**Figure 5:**
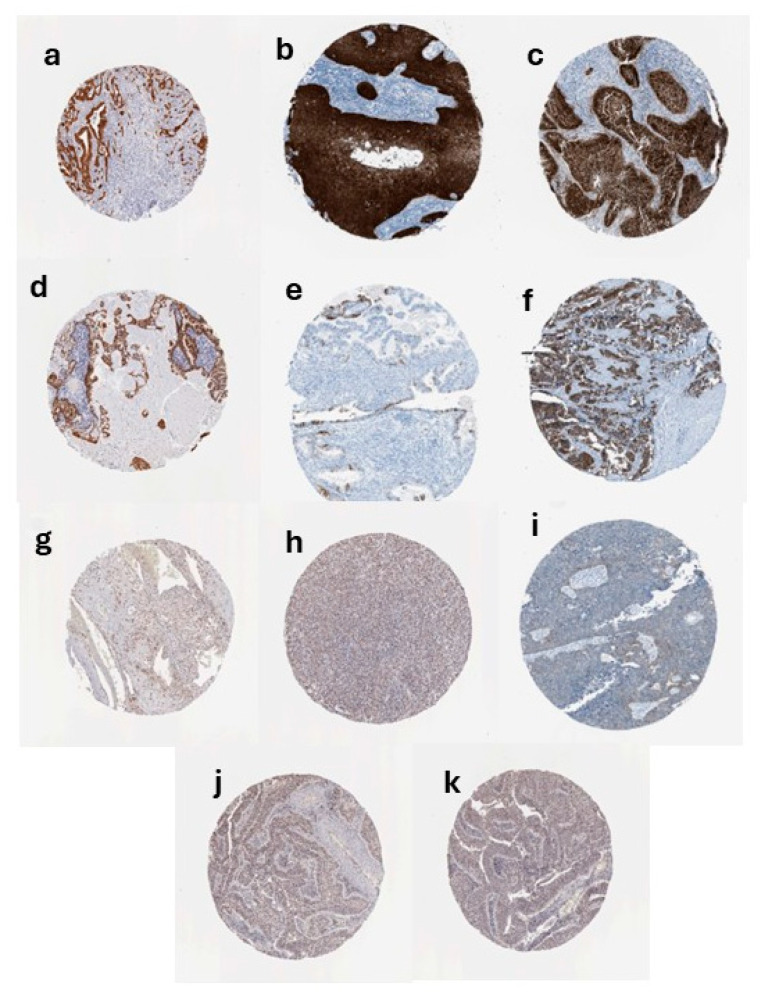
CDKN2A Immunohistochemistry in Cervical Cancer and Normal Cervix. Representative IHC staining of CDKN2A in cervical tissues (Human Protein Atlas). Strong expression: (**a**–**c**) CESC tumor tissues showing intense nuclear and cytoplasmic staining. Moderate expression: (**d**–**f**) CESC tumor tissues with moderate staining. Low expression: (**g**–**i**) CESC tumor tissues with weak staining. Normal tissues: (**j**,**k**) Normal cervical epithelium showing minimal/absent staining.

Analysis of Figure 5 indicates that CDKN2A protein expression is markedly increased in CESC compared with normal cervical epithelium, as demonstrated by immunohistochemistry data from the HPA. Tumor cores exhibit a range of positive staining intensities: Strong nuclear and cytoplasmic staining in neoplastic epithelial cells (a–c), Moderate staining (d–f), and Low/weak staining (g–i), while normal cervix shows minimal to absent staining (j–k). Staining in tumors is predominantly localized to the nuclei with concurrent cytoplasmic signal, and is most conspicuous in the carcinoma cell nests; stromal elements are largely negative. These protein-level findings corroborate the transcriptomic overexpression observed in CESC and support the feasibility of CDKN2A/p16^INK4a^ as a tissue biomarker in cervical cancer. p16^INK4a^ IHC is widely used in cervical pathology and has recognized diagnostic and prognostic utility in HPV-associated disease, aligning with our IHC observations [7].

**Figure 6:**
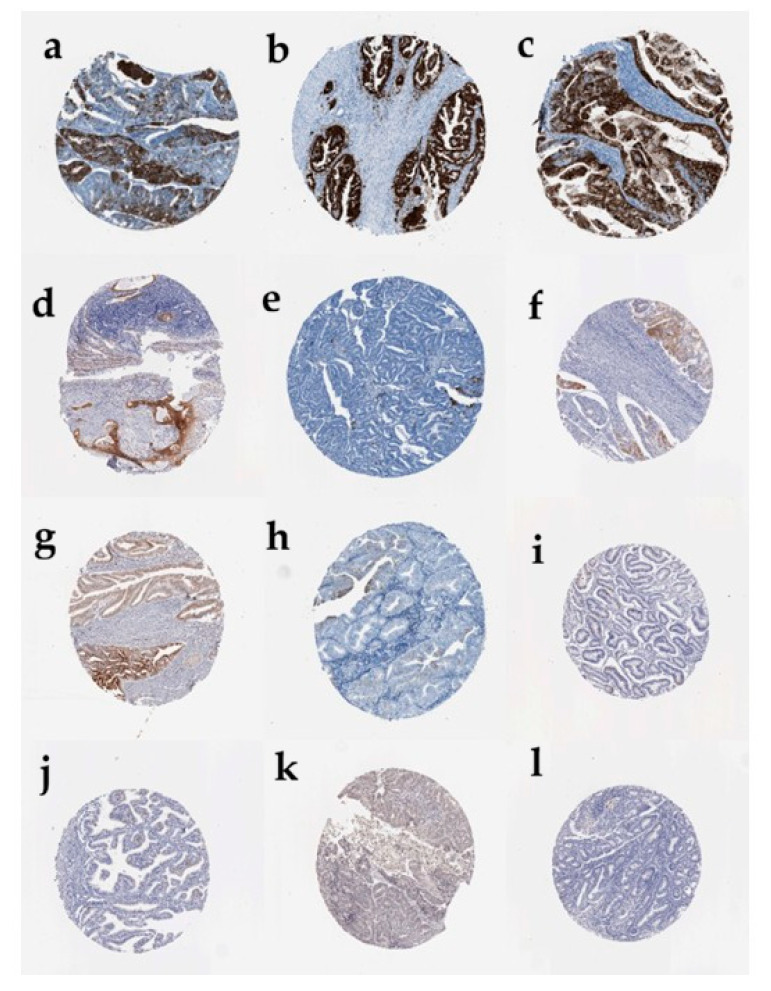
CDKN2A Immunohistochemistry in Endometrial Cancer and Normal Uterus. Representative IHC staining of CDKN2A in endometrial tissues (Human Protein Atlas). Strong expression: (**a**–**c**) UCEC tumor tissues showing strong nuclear and cytoplasmic staining. Moderate expression: (**d**–**f**) UCEC tumor tissues with moderate staining. Low expression: (**g**–**i**) UCEC tumor tissues with low/weak staining. Normal tissues: (**j**–**l**) Normal endometrial glandular epithelium with minimal staining.

The Human Protein Atlas’s immunohistochemical analysis has revealed a marked increase in CDKN2A protein expression in endometrial carcinoma (UCEC) tissues compared to normal endometrial epithelium. Tumor samples displayed a spectrum of staining intensities, with a subset of UCEC tissues showing strong nuclear and cytoplasmic CDKN2A positivity (a–c), while other tumor cores exhibited moderate (d–f) or low/weak staining (g–i). In contrast, normal endometrial glandular tissue showed minimal or absent CDKN2A staining (j–l), indicating very low basal protein expression in non-malignant uterine epithelium. The staining pattern in tumors was predominantly localized to the nuclei of neoplastic epithelial cells, with variable cytoplasmic involvement, while the surrounding stromal regions remained largely negative. This protein-level overexpression corroborates the transcriptomic findings in UCEC and underscores the potential of CDKN2A as a clinically relevant diagnostic and prognostic biomarker in endometrial cancer (Figure 6).

**Figure 7:**
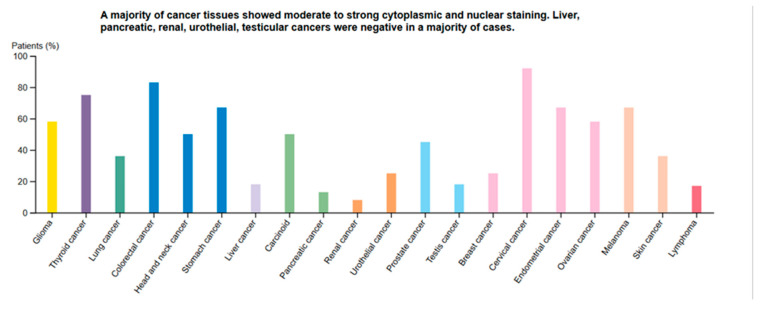
CDKN2A Protein Expression Across Cancers (HPA Pathology Atlas). Bar chart summary of CDKN2A protein expression across 17 cancer types, based on immunohistochemistry. The *y*-axis shows percentage of patient samples with moderate to strong cytoplasmic/nuclear staining. Cervical cancer (~90%) and Endometrial cancer (~65%) exhibited a high proportion of CDKN2A-positive cases, confirming elevated protein expression in gynecological malignancies.

To evaluate the distribution of CDKN2A protein expression across various tumor types, we examined immunohistochemical data from the Human Protein Atlas (HPA) Pathology Atlas (Figure 7). The bar chart summarizes the percentage of patient samples exhibiting moderate to strong cytoplasmic and nuclear staining for CDKN2A across 17 cancer types. The results indicated that CDKN2A was highly expressed in a majority of cervical (~90%) and endometrial (~65%) cancer tissues, confirming its consistent upregulation in gynecological malignancies. Elevated expression was also observed in colorectal, lung, thyroid, and head and neck cancers, with more than 50% of patient samples showing positive staining. In contrast, liver, pancreatic, renal, urothelial, and testicular cancers demonstrated low or absent CDKN2A staining in most cases, suggesting tissue-specific regulation of CDKN2A expression. Overall, these outcomes support the widespread yet variable expression of CDKN2A protein among human cancers, with particularly high prevalence in cervical and endometrial cancers, further validating its potential as a diagnostic and prognostic biomarker in gynecologic oncology.

**Figure 8:**
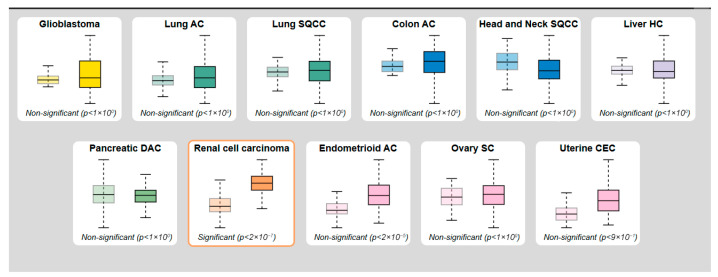
CDKN2A RNA Expression in Multiple Cancers (HPA/TCGA). Boxplot analysis of CDKN2A mRNA expression in tumor versus normal tissues across multiple cancer types, derived from RNA-seq data (TCGA) presented in the Human Protein Atlas. Significant overexpression was observed in renal cell carcinoma (*p* < 2 × 10^−7^). Expression differences in CESC (uterine cervical squamous cell carcinoma) and UCEC (endometrioid adenocarcinoma) were not statistically significant. These data emphasize tumor-specific variability and support the need for multi-level validation (RNA and protein) in gynecological cancers. In each panel, the left box represents normal tissue and the right box represents tumor tissue.

To further validate CDKN2A transcript levels across various malignancies, RNA sequencing data from the TCGA dataset integrated in the Human Protein Atlas (HPA) were analyzed (Figure 8). The boxplot analysis compared CDKN2A mRNA expression between tumor and corresponding normal tissues across multiple cancer types. Among the cancers examined, renal cell carcinoma exhibited a statistically significant increase in CDKN2A expression (*p* < 2 × 10^−7^), while other tumor types, including cervical squamous cell carcinoma (CESC) and endometrioid adenocarcinoma (UCEC), displayed non-significant expression differences (*p* > 0.05). Analysis of TCGA RNA-seq data accessed through the Human Protein Atlas indicated no statistically significant tumor–normal differences in CDKN2A transcript levels in either CESC or UCEC. The interpretation of this observation requires caution due to the limited availability of normal samples in TCGA-only comparisons, which compromises statistical power. Conversely, integrated analyses utilizing GEPIA, which merges TCGA tumor samples with GTEx normal tissues, have revealed markedly elevated CDKN2A expression patterns. Independent validation through GEO datasets has further corroborated the increased expression across various cohorts. In total, these findings imply that the differences observed across platforms are indicative of variations in cohort composition rather than biological contradictions. Consequently, conclusions are drawn from integrative multi-cohort evidence rather than relying on single-dataset comparisons alone.

**Figure 9:**
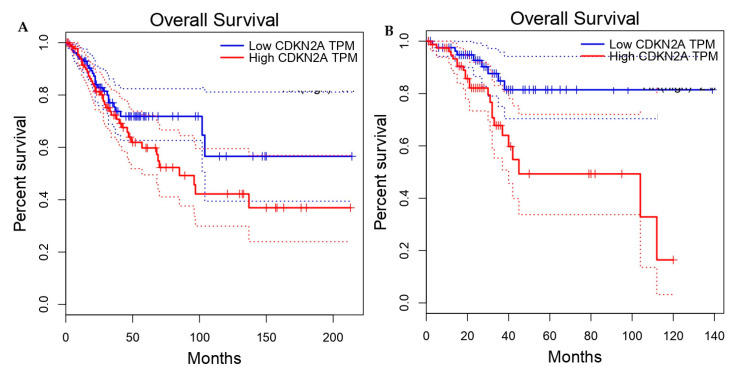
The Kaplan–Meier overall survival curves illustrate a comparison between high and low CDKN2A expression groups, as determined by median stratification. The red lines represent the high-expression cohorts, while the blue lines denote the low-expression cohorts. The shaded or dotted regions indicate 95% confidence intervals. Statistical significance was assessed using the log-rank test, and hazard ratios were calculated through Cox proportional hazards modeling. Log-rank *p* = 0.0055; HR = 2.9 (95% CI: 1.35–6.12). Notably, tumor-specific differences in prognostic association are evident between CESC (**A**) and UCEC (**B**).

**Figure 10:**
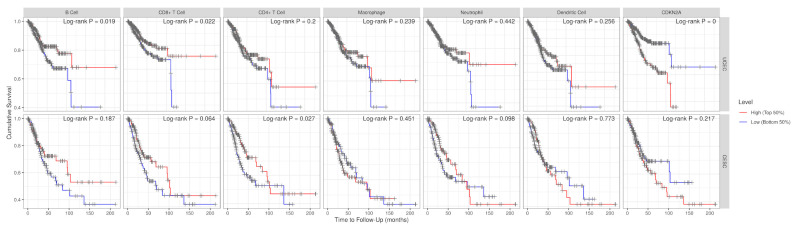
Kaplan–Meier overall survival curves showing exploratory associations between CDKN2A expression, immune cell infiltration levels, and patient survival in cervical squamous cell carcinoma (CESC) and uterine corpus endometrial carcinoma (UCEC). Analyses were performed using the TIMER web platform. Patients were stratified into high and low groups based on median expression or infiltration levels (top vs. bottom 50%). Red curves represent high-level groups and blue curves represent low-level groups. Statistical significance was assessed using the log-rank test, with corresponding *p*-values shown in each panel. Time is expressed in months. These plots are presented for visualization of immune-related trends, while cancer-specific prognostic conclusions were derived from independent GEPIA survival analyses.

Preliminary assessments of baseline survival were conducted using Kaplan–Meier stratification derived from GEPIA, based on median CDKN2A expression, to determine primary prognostic effects. Subsequent analyses utilizing TIMER were employed to investigate immune-contexture interactions affecting survival patterns. These analyses should be regarded as complementary mechanistic investigations rather than primary prognostic evidence. The Kaplan–Meier survival analysis, stratified by median CDKN2A expression levels, demonstrated distinct prognostic patterns across different tumor types. In CESC, increased CDKN2A expression did not show a statistically significant correlation with overall survival (log-rank *p* = 0.13; HR = 1.4), indicating a limited capacity for prognostic differentiation (Figure 9A). Conversely, in UCEC, a significant correlation was identified between elevated CDKN2A expression and reduced survival rates (log-rank *p* = 0.0055; HR = 2.9), highlighting the prognostic relevance that is contingent on the tumor context (Figure 9B). These results are consistent with the presence of tumor-specific regulatory mechanisms and emphasize the necessity of assessing CDKN2A within the molecular framework specific to each disease. Survival analyses demonstrated tumor-specific differences in the prognostic relevance of CDKN2A expression. In cervical squamous cell carcinoma (CESC), higher CDKN2A expression showed no statistically significant association with overall survival (log-rank *p* = 0.217; HR = 1.37, 95% CI 0.85–2.19). This lack of prognostic discrimination is biologically consistent with HPV-driven tumorigenesis, where p16 overexpression represents an early and nearly ubiquitous consequence of E7-mediated RB pathway inactivation. Because most tumor cells already exhibit maximal pathway activation, variation in CDKN2A expression is unlikely to stratify outcome. In contrast, uterine corpus endometrial carcinoma (UCEC) analysis revealed a significant association between elevated CDKN2A expression and poorer overall survival (log-rank *p* = 0.0023; HR = 3.13, 95% CI 1.44–6.76). This finding suggests that CDKN2A upregulation in UCEC reflects tumor-context-specific biological processes distinct from viral oncogenesis. Age-associated increases in p16 expression and links to cellular senescence pathways may contribute to enrichment within aggressive molecular subtypes or stress-induced checkpoint activation states. Accordingly, prognostic interpretation of CDKN2A must remain tumor-context dependent and strictly aligned with statistical evidence (Figure 10).

## 4. Validation

**Figure 11:**
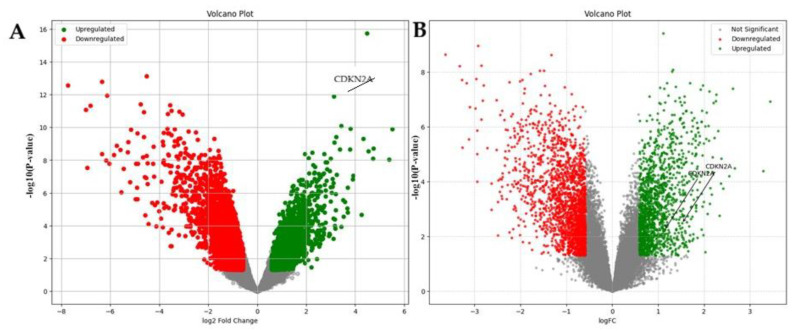
Volcano plots showing differential gene expression in (**A**) cervical cancer dataset GSE9750 and (**B**) endometrial cancer dataset GSE63678. Each dot represents an individual gene; green dots indicate upregulated, red dots indicate downregulated genes, and grey dots represent non-significant genes. CDKN2A is highlighted among the significantly upregulated genes (*p* < 0.05).

To validate CDKN2A differential expression at the transcriptomic level, we examined two independent GEO microarray datasets related to cervical cancer (GSE9750; Figure 11A) and endometrial cancer (GSE63678; Figure 11B). In both datasets, CDKN2A emerged as one of the significantly upregulated genes in tumor samples compared to normal tissues, as indicated by its position in the upper right quadrant of the volcano plots. The distinct separation of CDKN2A from non-significant genes, along with its high log_2_ fold-change and low *p*-value, underscores robust transcriptional activation in both cancer types. These findings validate the overexpression of CDKN2A in independent cohorts and affirm its consistency as a tumor-associated gene signature in gynecologic malignancies.

CDKN2A demonstrated consistent tumor-associated dysregulation across transcriptomic and proteomic platforms, with particularly pronounced effects at the protein level, as evidenced by strong tumor-specific staining in immunohistochemistry. RNA-based findings varied among cohorts, with significant elevation observed in integrated GEPIA and GEO analyses, whereas TCGA-only comparisons did not yield statistical significance. These findings highlight the necessity of multi-platform interpretation when assessing biomarker behavior. The IHC staining showed strong p16^INK4a^ presence in tumor tissues, while normal tissues had little or no staining, highlighting its specific overexpression in tumors. These findings confirm that CDKN2A is a reliable marker to tell apart cancerous from non-cancerous gynecologic tissues. This matches current medical practice, where p16^INK4a^ staining helps diagnose HPV-related cervical cancer. For prognosis, CDKN2A showed different patterns depending on the cancer type. Higher CDKN2A levels were linked to worse survival in CESC, suggesting a negative outlook for cervical cancer. However, its impact on UCEC survival was limited or neutral, possibly due to differences in tumor types, molecular subtypes, or immune environments.

In short, CDKN2A is a strong diagnostic marker for cervical and endometrial cancers, backed by consistent data. While it is a clear prognostic marker in CESC, it is less so in UCEC, showing the need to consider specific tumor contexts and biological factors that might affect protein levels.

Diagnostic Classification Performance of CDKN2A in Independent GEO Cohorts:

To formally assess the discrimination between tumor and normal samples, we conducted a cross-validated ROC analysis utilizing independent GEO datasets. In the CESC dataset GSE9750 (*n* = 65; 41 tumor, 24 normal), CDKN2A exhibited exceptional diagnostic discrimination, achieving a cross-validated AUC of 0.982 (95% CI: 0.946–1.000). At the Youden-optimal threshold, sensitivity was 95.1%, specificity was 100%, and balanced accuracy was 0.976. The ROC curve indicated nearly complete separation between tumor and normal samples, corroborating the robust classification performance. Conversely, in the UCEC dataset GSE63678 (*n* = 35; 18 tumor, 17 normal), CDKN2A displayed moderate discriminatory capability, with a cross-validated AUC of 0.761 (95% CI: 0.587–0.909). Sensitivity and specificity at the optimal threshold were 77.8% and 70.6%, respectively, with a balanced accuracy of 0.742. The broader confidence interval reflects the smaller cohort size and increased heterogeneity. These results provide quantitative evidence supporting CDKN2A as a potent diagnostic marker in CESC and a potential diagnostic adjunct in UCEC, with performance contingent on the cohort.

## 5. Discussion

This research offers a thorough multi-omics assessment of CDKN2A (p16^INK4a^) expression in cervical squamous cell carcinoma (CESC) and uterine corpus endometrial carcinoma (UCEC). By integrating transcriptomic, proteomic, and clinical outcome data from a variety of independent sources, we confirm that CDKN2A is consistently dysregulated in both types of cancer, with particularly pronounced tumor-specific protein expression. These findings endorse CDKN2A as a dependable diagnostic biomarker in gynecologic oncology, although its prognostic value may vary depending on the context. Further pan-cancer analysis revealed extensive CDKN2A upregulation across a range of tumor types, corroborating previous TCGA-based studies that identify p16^INK4a^ activation as a prevalent molecular characteristic of malignant transformation, linked to the disruption of the RB–E2F cell-cycle checkpoint [13]. In the context of cervical cancer, the overexpression of CDKN2A is mechanistically linked to high-risk human papillomavirus (HPV) infection. The HPV E7 oncoprotein interacts with and facilitates the degradation of the retinoblastoma (RB) tumor suppressor protein, resulting in deregulated E2F transcriptional activity and a compensatory increase in p16^INK4a^ expression. This molecular feedback mechanism explains the characteristic strong and diffuse nuclear and cytoplasmic p16 staining observed in HPV-driven cervical neoplasia [14]. Our results align with previous molecular and histopathological studies that demonstrate a close correlation between p16 overexpression, RB loss, and high-risk HPV infection in cervical tumors [4,14] Consequently, p16^INK4a^ immunohistochemistry is widely recognized as a surrogate marker for transforming HPV infection and is routinely used in diagnostic practice. The diagnostic importance of p16^INK4a^ in cervical pathology is well-established in international guidelines. The College of American Pathologists (CAP) and the American Society for Colposcopy and Cervical Pathology (ASCCP) advocate for p16 immunostaining in the classification of high-grade squamous intraepithelial lesions and in resolving morphologically ambiguous cases. Extensive clinicopathological studies report p16 positivity in over 95–99% of CIN3 lesions and invasive cervical carcinomas, with minimal expression in benign cervical epithelium. The strong protein-level expression observed in our CESC cohort is thus consistent with current clinical standards and underscores the diagnostic reliability of CDKN2A in HPV-associated cervical disease.

In contrast to cervical cancer, uterine corpus endometrial carcinoma (UCEC) is largely independent of human papillomavirus (HPV) involvement. Meta-analyses and molecular investigations reveal that only a minor fraction of endometrial carcinomas contain HPV DNA, indicating that HPV is not a significant etiological factor in this malignancy [5]. Instead, UCEC is primarily driven by hormonal dysregulation and recurrent genetic alterations in PTEN, PIK3CA, KRAS, CTNNB1, and TP53, which define distinct molecular subtypes [5]. Despite this fundamental etiological distinction, our analyses identified significant overexpression of CDKN2A at the protein level in a considerable subset of UCEC samples. These findings align with previous reports of p16^INK4a^ overexpression in high-grade serous and dedifferentiated endometrial carcinomas, where it is believed to result from oncogenic stress, RB pathway disruption, or epigenetic deregulation rather than viral oncogenesis [15,16]. While p16 overexpression in cervical cancer is classically attributed to HPV-mediated RB inactivation, elevated CDKN2A expression may also arise through HPV-independent mechanisms relevant to UCEC biology. CDKN2A functions as a key mediator of cellular senescence, and its expression increases with aging and stress-induced chromatin depression, leading to CDK4/6 inhibition and cell-cycle arrest [17]. Oxidative stress and DNA-damage signaling can further induce p16 independently of viral pathways [18], linking its expression to genomic instability and cellular stress responses. In addition, oncogene activation or tumor-suppressor loss may trigger senescence programs that elevate p16 as an antiproliferative safeguard. These mechanisms collectively provide biological plausibility for p16 elevation in HPV-negative malignancies and support interpreting CDKN2A upregulation in UCEC as a marker of cell-cycle dysregulation rather than viral oncogenesis [18].

In addition, consideration should be given to the molecular heterogeneity of endometrial cancer, particularly the “p53-mutant” or serous-like phenotype. Uterine serous carcinomas frequently exhibit diffuse p16 overexpression despite the absence of HPV infection, demonstrating that CDKN2A activation in these tumors reflects HPV-independent oncogenic processes rather than viral signaling [19].

Immunophenotypic studies further show that tumors displaying mutation-type p53 staining together with strong p16 positivity are strongly associated with serous histology, indicating distinct pathogenic mechanisms compared with endometrioid subtypes [19]. This phenotype is consistent with the high prevalence of TP53 mutation and protein accumulation observed in serous endometrial carcinoma [19]. Prior experimental studies have shown that oncogenic stress and CDK4/6–pRB axis disruption can induce p16 expression; however, our analysis cannot confirm these mechanisms and instead demonstrates expression associations consistent with such models [20].

This suggests that p16 induction represents a convergent molecular response to cell-cycle dysregulation shared by both HPV-dependent and HPV-independent gynecologic tumors. Analyzing CDKN2A transcript expression across various datasets necessitates consideration of the cohort structure specific to each platform. Comparisons limited to TCGA, as derived from HPA, revealed non-significant differences between tumor and normal samples, likely due to the limited representation of normal samples. Conversely, integrated analyses from GEPIA and independent validation through GEO consistently indicated elevated expression patterns. These findings suggest that the apparent discrepancies are attributable to statistical limitations rather than biological contradictions. Therefore, conclusions are drawn from convergent evidence across multiple cohorts rather than inferring post-transcriptional regulation without experimental validation. In contrast, immunohistochemistry showed strong and consistent p16 protein accumulation in tumor tissues. The apparent discrepancy between transcriptomic datasets requires cautious interpretation. The lack of significant CDKN2A mRNA differences observed in TCGA-derived comparisons should not be interpreted as evidence of biological discordance, as these analyses rely on a very limited number of normal samples for certain tumor types, resulting in reduced statistical power. In contrast, GEPIA integrates TCGA tumor data with GTEx normal tissue cohorts, providing a more representative baseline and likely explaining the significant upregulation observed in our analysis. Independent validation using GEO datasets further supports transcriptional elevation of CDKN2A across platforms. Collectively, these findings indicate that differences across analytical outputs are primarily attributable to dataset composition and sampling limitations rather than post-transcriptional regulatory mechanisms. Accordingly, interpretation of CDKN2A mRNA expression in this study is based on integrated and independently validated cohorts, while TCGA-only comparisons are acknowledged as methodologically constrained. These findings highlight the necessity of integrating proteomic data with transcriptomic analyses when evaluating candidate biomarkers for clinical use. Survival analyses have identified tumor-type-specific variations in the prognostic significance of CDKN2A expression. The survival curves, generated through explicit median-based stratification, further underscore the context-dependent prognostic behavior of CDKN2A, revealing non-significant discrimination in HPV-driven cervical tumors and significant risk stratification in endometrial carcinoma. In cervical squamous cell carcinoma (CESC), elevated CDKN2A expression showed no statistically significant association with overall survival (log-rank *p* = 0.217; HR = 1.37, 95% CI 0.85–2.19). This limited prognostic discrimination is biologically consistent with HPV-driven tumorigenesis, in which p16 overexpression represents an early and nearly ubiquitous consequence of E7-mediated RB pathway inactivation [21,22]. Because most cervical tumor cells already exhibit maximal pathway activation, variation in CDKN2A expression is unlikely to stratify clinical outcome, reinforcing its role as a diagnostic rather than prognostic biomarker.

In contrast, uterine corpus endometrial carcinoma (UCEC) demonstrated a significant association between elevated CDKN2A expression and poorer overall survival (log-rank *p* = 0.0023; HR = 3.13, 95% CI 1.44–6.76). This suggests that CDKN2A upregulation in endometrial cancer reflects tumor-context-specific biological processes distinct from viral oncogenesis. Age-associated increases in p16 expression and links to cellular senescence pathways may contribute to enrichment within aggressive molecular subtypes or reflect stress-induced checkpoint activation. Collectively, these findings underscore that CDKN2A should not be interpreted as a universal prognostic marker; rather, its clinical implications are strongly tumor-dependent and must be evaluated in the context of underlying molecular etiology (Figure 9).

The context-dependent nature of CDKN2A function across tumor types further underscores the importance of interpreting this biomarker within its molecular and tissue-specific landscape. While CDKN2A overexpression in gynecologic malignancies reflects cell-cycle checkpoint activation and stress-response signaling, melanoma represents a contrasting paradigm in which CDKN2A is frequently inactivated or deleted. Germline mutations in CDKN2A constitute a major hereditary risk factor for melanoma, and loss of its function enables escape from oncogene-induced senescence triggered by activating BRAF mutations. These opposing biological behaviors highlight the role of p16 as a cellular stress sensor: overexpressed when regulatory pathways are overwhelmed and absent when checkpoint control is eliminated. Incorporating this perspective reinforces the view that CDKN2A cannot be interpreted as a uniform biomarker across cancers but must be evaluated within tumor-specific molecular context [23].

Independent validation using GEO microarray datasets (GSE9750 for CESC and GSE63678 for UCEC) has confirmed the significant upregulation of CDKN2A in both malignancies, thereby supporting the reproducibility of our findings across various platforms. The alignment observed between RNA-based validation and protein-level immunohistochemistry enhances the credibility of CDKN2A as a diagnostic marker for distinguishing between malignant and non-malignant gynecologic tissues. Clinically, these findings extend the role of p16 beyond HPV-associated cervical neoplasia, indicating its potential utility in the stratification of endometrial tumors, especially high-grade subtypes that may be challenging to differentiate morphologically [4,6]. From a translational standpoint, these results highlight the significance of CDKN2A as a marker of cell-cycle dysregulation in gynecologic oncology. In cervical cancer, p16^INK4a^ immunohistochemistry remains a vital diagnostic tool for confirming HPV-driven lesions. In endometrial carcinoma, evaluating CDKN2A expression may help identify biologically aggressive subgroups and could complement molecular classification schemes [24]. Moreover, as p16 overexpression indicates disruption of the CDK4/6–RB axis, These findings emphasize the significance of CDKN2A as an indicator of cell-cycle dysregulation. Although earlier experimental research suggests that targeting the CDK4/6 pathway or modulating the immune system may have therapeutic potential, the current study does not investigate treatment responses. Therefore, these implications should be considered as generating hypotheses rather than providing conclusions with direct clinical application. In summary, this research delivers a comprehensive multi-omics analysis of CDKN2A (p16^INK4a^) in the context of both HPV-associated CESC and HPV-independent UCEC. By synthesizing data from transcriptomic, proteomic, clinical, and independent validation sources, we identify tumor-specific patterns of CDKN2A dysregulation with unique diagnostic implications. The findings are further substantiated by formal diagnostic classification analysis using independent GEO cohorts. In CESC, CDKN2A demonstrates outstanding tumor–normal discrimination, evidenced by a cross-validated AUC of 0.982, along with high sensitivity and specificity, achieving near-complete separation between malignant and normal tissues. This performance is consistent with its recognized role as a surrogate marker for HPV-mediated RB pathway inactivation, underscoring its robust diagnostic utility in cervical pathology. In contrast, CDKN2A shows moderate and cohort-dependent discrimination in UCEC (AUC = 0.761), indicative of greater molecular heterogeneity and HPV-independent oncogenic processes. Although CDKN2A expression is elevated at the protein level in a significant subset of UCEC cases, its classification performance suggests it should be considered a diagnostic adjunct rather than a standalone biomarker in endometrial carcinoma. Overall, these results highlight the importance of interpreting CDKN2A expression within the specific tumor context. In CESC, CDKN2A upregulation is a biologically coherent outcome of HPV-driven RB disruption, providing strong tumor–normal discriminatory power. In UCEC, CDKN2A overexpression may reflect alternative oncogenic stress pathways or senescence-associated processes independent of HPV. However, because this study is based on transcriptomic and proteomic association data, these mechanistic interpretations remain hypothesis-generating and require direct functional validation. Importantly, this study represents an integrative bioinformatics analysis of publicly available datasets. While the observed expression patterns are biologically consistent with established RB pathway biology and senescence-associated signaling, direct mechanistic conclusions cannot be drawn without experimental validation elevated in integrated cohorts.

## 6. Conclusions

In summary, this study provides an integrated multi-omics evaluation of CDKN2A (p16^INK4a^) in cervical squamous cell carcinoma (CESC) and uterine corpus endometrial carcinoma (UCEC). Across transcriptomic and proteomic datasets, CDKN2A showed consistent tumor-associated upregulation, with strong protein-level expression in approximately 90% of CESC and 65% of UCEC cases, while normal tissues showed minimal staining. Importantly, formal diagnostic classification analysis in independent GEO cohorts demonstrated excellent tumor–normal discrimination in CESC (AUC = 0.982) and moderate discrimination in UCEC (AUC = 0.761). These findings quantitatively support CDKN2A as a strong diagnostic marker in cervical cancer and as a diagnostic adjunct in endometrial carcinoma, where performance appears more cohort-dependent. Prognostic associations were tumor-specific. In CESC, CDKN2A upregulation reflects HPV-mediated RB pathway disruption and showed limited survival stratification, consistent with its role as a diagnostic marker. In contrast, UCEC demonstrated context-dependent prognostic patterns, likely reflecting HPV-independent oncogenic stress mechanisms. Although this study relies on publicly available datasets and in silico analyses, the consistent results across independent transcriptomic and proteomic platforms strengthen the validity of the findings. Overall, CDKN2A should be interpreted within its tumor-specific biological context, with clear diagnostic strength in CESC and supportive but more moderate utility in UCEC.

## Data Availability

The datasets analyzed in this study are publicly available. Transcriptomic and clinical data were obtained from The Cancer Genome Atlas (TCGA), Gene Expression Profiling Interactive Analysis (GEPIA), TIMER2.0, UALCAN, and the Human Protein Atlas (HPA). Independent validation datasets were retrieved from the Gene Expression Omnibus (GEO) under accession numbers GSE9750 and GSE63678. All data used in this study are available from the corresponding public repositories.

## Supplementary Materials

The following supporting information can be downloaded at: https://www.mdpi.com/article/10.3390/genes17030281/s1, Figure S1. Pan-cancer expression analysis of CDKN2A across multiple tumor types using the GEPIA database (TCGA and GTEx data). Boxplots compare CDKN2A expression levels between tumor tissues (red) and corresponding normal tissues (gray). Significant overexpression is observed in several cancer types, including BLCA, BRCA, CESC, COAD, HNSC, KICH, KIRC, LIHC, LUAD, LUSC, PRAD, READ, STAD, THCA, and UCEC (*p* < 0.05). These results indicate widespread dysregulation of CDKN2A in solid tumors.; Figure S2. Refined pan-cancer expression profile of CDKN2A after filtering to include only cancer types with significant upregulation. Boxplots illustrate CDKN2A expression in tumor tissues (red) compared with matched normal tissues (gray). The selected cancer types (BLCA, BRCA, CESC, COAD, HNSC, KICH, KIRC, KIRP, LIHC, LUAD, LUSC, READ, STAD, and UCEC) consistently exhibit higher median expression in tumors, confirming a recurrent pattern of CDKN2A activation in malignancies characterized by cell-cycle dysregulation. Figure S3. Kaplan–Meier survival analysis comparing overall survival between early-stage and late-stage cancer patients. Survival probability is plotted against time (days), with tick marks indicating censored observations. No statistically significant difference in survival was observed between early and late stage groups (log-rank *p* = 0.57). Numbers at risk are shown below the plot. File S1. Complete Python code used for GEO data processing, differential expression analysis, ROC/AUC calculation, cross-validation, and figure generation.
