# Supplementary material for: Multi-Omics Analysis of CDKN2A (p16INK4a) in Cervical Carcinoma in the Context of Human Papillomavirus and in Endometrial Carcinoma
Source: Genes (Basel). 2026 Feb 27;17(3):281. doi: 10.3390/genes17030281 (PMC13025831; doi:10.3390/genes17030281)
Supplement: Supplementary file 1 [file genes-17-00281-s001.zip › genes-4161991-supplementary.pdf]

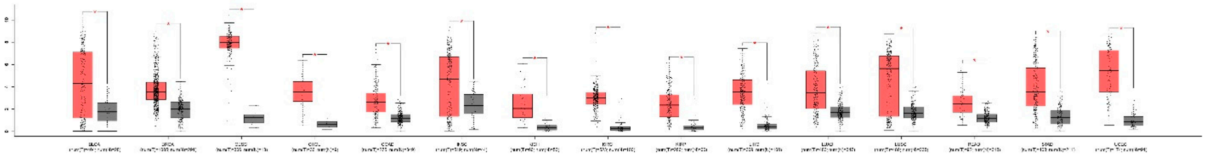

Supplementary Figure S1.

Pan-cancer expression analysis of **CDKN2A** across multiple tumor types using the GEPIA database (TCGA and GTEx data). Boxplots compare CDKN2A expression levels between tumor tissues (red) and corresponding normal tissues (gray). Significant overexpression is observed in several cancer types, including BLCA, BRCA, CESC, COAD, HNSC, KICH, KIRC, LIHC, LUAD, LUSC, PRAD, READ, STAD, THCA, and UCEC ($p < 0.05$). These results indicate widespread dysregulation of CDKN2A in solid tumors.

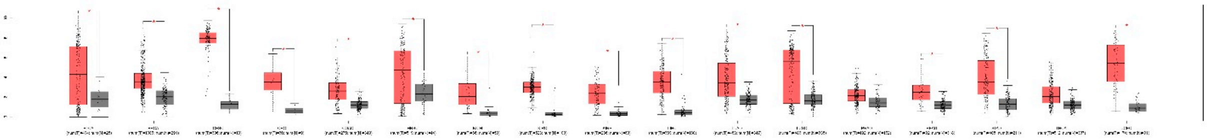

Supplementary Figure S2.

Refined pan-cancer expression profile of **CDKN2A** after filtering to include only cancer types with significant upregulation. Boxplots illustrate CDKN2A expression in tumor tissues (red) compared with matched normal tissues (gray). The selected cancer types (BLCA, BRCA, CESC, COAD, HNSC, KICH, KIRC, KIRP, LIHC, LUAD, LUSC, READ, STAD, and UCEC) consistently exhibit higher median expression in tumors, confirming a recurrent pattern of CDKN2A activation in malignancies characterized by cell-cycle dysregulation.

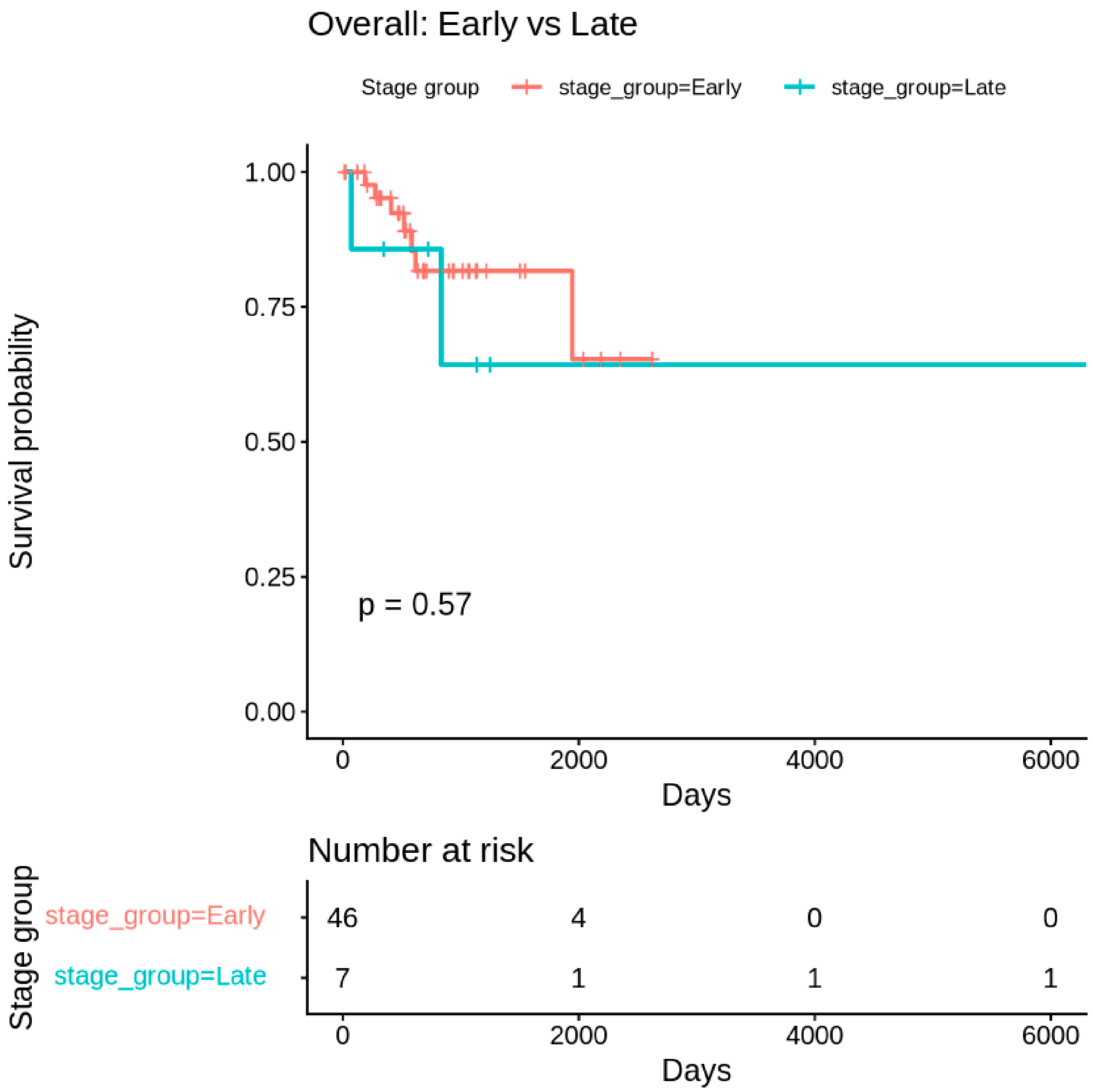

Supplementary Figure S3.

Kaplan–Meier survival analysis comparing overall survival between **early-stage** and **late-stage** cancer patients. Survival probability is plotted against time (days), with tick marks indicating censored observations. No statistically significant difference in survival was observed between early and late stage groups (log-rank p = 0.57). Numbers at risk are shown below the plot.
